# Structural insights into thraustochytrid-specific lipases using alphafold to identify the role of GXSXG motif

**DOI:** 10.1186/s12896-025-00972-8

**Published:** 2025-05-05

**Authors:** Iqra Mariam, Ulrika Rova, Paul Christakopoulos, Leonidas Matsakas, Alok Patel

**Affiliations:** Biochemical Process Engineering, Division of Chemical Engineering, Department of Civil, Environmental, and Natural Resources Engineering, Luleå University of Technology, Luleå, SE-971 87 Sweden

**Keywords:** Alpha fold, Ligand docking, Lipase, Phylogenomics, Thraustochytrids

## Abstract

**Background:**

Triacylglycerol lipases (E.C. 3.1.1.3) are serine hydrolases, universally present in animals, plants and microbes and are an integral part of lipid metabolism. They are industrially relevant enzymes that cleave ester bonds of triacylglycerides to release free fatty acids and glycerol. Thraustochytrid *Aurantiochytrium limacinum* SR21 has previously been reported to utilize 120 g L^− 1^ of oil substrate. Previously, thraustochytrid specific lipases was reported that allowed the microbe to thrive on oil substrate, however the structural characteristics of these enzymes remain undetermined.

**Results:**

In this study, we identified nearly 30 genes that encode TAG lipases with Lipase_3 domain, allowing the marine microbe to thrive on oil substrate. The lipases were predicted to localize at several subcellular compartments such as extracellular (31293), membrane-bound and cytosolic. Phylogenomic analysis revealed that lipases from thraustochytrids form distinct clades, diverging significantly from the well-characterized lipases from yeast *Yarrowia lipolytica*. Motif enrichment analysis confirmed the presence of the conserved ‘GXSXG’ motif in all lipases, where serine serves as the catalytic residue. Notably, histidine (H) or tyrosine (Y) was found at the second position of the motif *in A. limacinum* SR21 lipases 34357 (cytosolic) and 31293 (extracellular) respectively, suggesting functional differences. Docking analysis with tripalmitoylglycerol (4RF) revealed lower binding energy (ΔG = -5.7 kcal/mol) for cytoplasmic lipase 34357, indicating a stronger ligand interaction compared to ΔG = -3.4 kcal/mol for the extracellular lipase 31293. This suggests that substituting histidine for tyrosine in the active site affects lipase catalytic efficiency and substrate specificity.

**Conclusions:**

Our study provides novel insights regarding the structure and ligand binding affinities for thraustochytrid specific lipases which are diversified attributed to the heterogeneity within the catalytic triads. In conclusion, we hypothesize that differential localization and higher binding efficiency of thraustochytrid specific lipases allow the microbe to efficiently utilize oil substrates. These thraustochytrid-specific lipases are potential candidates for commercialization as large-scale production of thraustochytrids can be achieved sustainably by cultivating on sustainable substrates and these enzymes are highly efficient and robust.

**Supplementary Information:**

The online version contains supplementary material available at 10.1186/s12896-025-00972-8.

## Background

Lipases (EC 3.1.1.3) are serine hydrolases containing α/β hydrolase folds that hydrolyze complex triacylglycerols (TAGs) to release free fatty acids and glycerol moieties [1]. Lipases are industrially relevant enzymes which are currently used as additives in detergent, pharmaceutical sector, paper and food processing as well as biocatalyst in production of biodiesel and chemicals [2]. In 2024, the size of the global lipase market was USD 606.80 million, with North America having the largest market share of 39.65%. The market is anticipated to expand at a compound annual growth rate (CAGR) of 6.28% from USD 643.63 million in 2025 to USD 985.54 million by 2032^i^. Attributed to their significant role in physiological processes such as fat metabolism, these enzymes are universally present in plants, animals and microbes [3]. In comparison to plants and animals, microbes undergo rapid evolution, have high genomic variation and can be easily cultivated and modified. Because of their wide range of catalytic activities, high yield production, ease of genetic manipulation, lack of seasonal fluctuations, consistent supply, greater stability, safety, and convenience, as well as the extremely high growth rate of microorganisms in economically viable media, microbial lipases are more valuable than those derived from plants or animals [4]. Among microbes, bacteria, yeast and fungi were reported to produce lipases, which are secreted in the extracellular environment and allow them to degrade oil substrates, thus providing competitive advantages over other microbes [5]. Identified bacterial hosts for lipase production include *Bacillus*, *Pseudomonas*, *Staphylococcus*, and *Burkholderia* [6, 7]. Lipases derived from fungus are relatively more stable, highly specific and secretory and therefore employed in various industrial processes. Major lipase producing fungal species include *Thermomyces lanuginosus*, *Rhizopus oryzae*, *Aspergillus niger*, *Candida* sp. and *Yarrowia lipolytica* [5]. Chemical properties such as thermostability, activity in varying range of pH, temperature, salinity and organic solvents are the major selection criterion for microbial lipase production. Thus, alternative extremophile microbes are frequently studied for production of lipases.

Thraustochytrids are marine heterotrophic microbes that are leading producers of omega-3 enriched oil. These microbes can be cultivated in various sustainable carbon sources such as biodiesel-derived glycerol, forest-biomass hydrolysates and tolerate high salinity i.e., up to 30 g L^− 1^ [8]. Several thraustochytrid species such as *Thraustochytrium* sp., *Aurantiochytrium *sp*.* PKU#Sed1, *Schizochytrium *sp*.* #Mn4, *Thraustochytrium *sp*.* #SW1, *and Thraustochytrium *sp*.* #SW2 were found to have high lipase activity [9]. Previous reports from our group found that thraustochytrid *Aurantiochytrium limacinum* SR21 can utilize approximately 120 g L^− 1^ of waste cooking oil as the sole carbon source to produce docosahexaenoic acid (DHA). This highlighted the efficient capability of *A. limacinum* SR21 to hydrolyze TAG present in the oil, further suggesting the presence of extracellular lipases [10]. Additionally, Ishibashi et al. found a lipase encoding protein (protein ID:145138) in *A. limacinum* that possess fungus like lipase (Lipase 3) domain, which is secreted in the media and enables the microalga to utilize triolein (5mM) [11]. However, the structural characteristics of these extracellular lipases as well as the catalytic variation among the lipases is not studied for thraustochytrid. In this context, we retrieved the sequences of proteins possessing lipase 3 domain (PF01764) of *A. limacinum* SR21 and evaluated their evolutionary distinctness using phylogenomics. Sequence analysis was performed for these proteins, and they were found to have distinct patterns of motifs and were enriched in various subcellular localizations. Further, structure was predicted using AlphaFold for lipases with varying motifs and their protein-ligand interaction was computed. Overall, our data corroborate the findings and demonstrate the higher catalytic efficiency of lipase with G**H**SXG motif as compared to G**Y**SXG, and suggest that thraustochytrid-specific lipases can be further engineered and are suitable for commercialization.

## Methods

### Gene retrieval & phylogenetic analysis

Protein sequences of *A. limacinum* SR21 lipases with lipase 3 domain (Pfam: PF01764) were retrieved from Joint Genome Institute (JGI) genome portal [12]. Sequences for lipases in *Yarrowia lipolytica* and other thraustochytrids were obtained from UniProt using taxonomic identifiers (4952, 284591 and 2699528) [13]. InterProScan was used to further check the presence of lipase domain in sequence retrieved from UniProt [14]. Protein sequences were aligned using ClustalW, and a phylogenetic tree was constructed using MEGA X software to comprehend the evolutionary relationships among the proteins using both neighbor-joining (N-J) method and maximum likelihood with a bootstrapping value of 1000 [15]. The evolutionary distances were computed using the Jones–Taylor–Thornton (JTT) matrix-based method as described previously [16].

### Prediction of subcellular localization and motif enrichment

In silico predictions for protein localization were performed using WoLF PSORT [17] and DeepLOC 2.0 [18]. SignalP 5.0 [19] was used for prediction of secretory signals whereas TMHMM 2.0 [20] was used for prediction of transmembrane domain in the protein. Motif prediction for the protein sequences was performed using the MEME suite 5.5.4 [21]. Parameters used for the motif prediction consisted of number of sites, 2–600; number of repetitions, 0–1 per sequence; width limit, 6–50; and maximum number of motifs, up to 3.

### Structure analysis

Alphafold2 was used for template-dependent prediction of structure for extracellular lipase (31293) and cytoplasmic lipase (34258) [22]. The crystal structure of Lip2 lipase from *Yarrowia lipolytica* (PDB ID: 3O0D) at 1.7 Å resolution in its closed conformation was used as the template for structure prediction [23]. The structure was predicted with default parameters using template mode, num_recycle = 3 and MMSeq2 as msa_mode. The predicted structure with the highest confidence (pLDDT and predicted aligned error (PAE)) was further refined using GalaxyRefine with default parameters (iterations = 5, side chain optimization, mild backbone perturbations and relaxation and energy minimization) [24]. Protein structure was visualized and evaluated using ChimeraX [25]. Prosa web servers were used to estimate the protein folding energy scores for the modeled structures by uploading the PDB files obtained after five rounds of refinement using GalaxyRefine [26]. PROCHECK was used for predicting the stereochemical quality of modeled protein structure by analyzing residue-by-residue geometry and overall structure geometry [27].

### Ligand binding prediction

Ligand binding site was predicted for the protein structure using PrankWeb that is based on the machine learning algorithm P2Rank [28]. Further, the ligand tripalmitoylglycerol (PDB: 4RF) was docked on both the lipases using AutoDock Vina in the predicted binding sites and DockThor, and futher docking parameters were computed [29, 30]. Further, the interaction between the protein and ligand was visualized using Discovery Studio and PLIP web interface [31–33].

## Results and discussion

### Phylogenomic analysis of proteins with lipase 3 domain and motif enrichment

Lipases are ubiquitously present in both prokaryotes and eukaryotes which allow them to utilize intracellularly accumulated TAG for energy generation and also allow them to thrive on fatty acid substrates [34, 35]. Attributed to these multi-spatial roles, these hydrolases are present in various subcellular localization in microbial hosts. Among eukaryotic hosts the non-pathogenic yeast; *Y. lipolytica* is well characterized for production of lipases. First secretory lipase was reported in 1948 for these microbes, soon after which multiple cell-wall bound lipases were identified [36, 37]. The cell-bound lipases differ from the extracellular lipase in various aspects and did not require oleic acid as a stabilizer-activator [38]. To identify the sequence similarity and conservation among lipases from thraustochytrids and *Y. lipolytica*, protein sequences with lipase 3 domain were retrieved from UniProt database for the yeast and thraustochytrid species *Aplanochytrium stocchinoi*,* Mucochytrium quahogii* and *Hondaea fermentalgiana*. Additionally, our previous transcriptomic datasets have identified 30 genes encoding to lipase 3 domain containing proteins in *A. limacinum* SR21 which was used for analysis [39]. Subcellular localization for these lipases were predicted using multiple computational tools (Table 1). *A. limacinum* SR21 was found to possess five secretory lipases with a secretory signal as predicted using SignalP and DeepLoc tools. However, among these proteins 150126 was predicted to be localized in mitochondria using WolfPsort. Aurli_150126 was also found to possess one transmembrane domain, which further rule-out its classification into secretory lipases. Additionally, 145138 was found to have no secretory signal and possess a transmembrane domain (predicted using THMM). Ishibashi et al., has reported that 145138 is a thraustochytrid-specific secretory protein which does not have a secretory signal and belong to type-ΙΙ transmembrane protein [11].

**Table 1:** Subcellular localization prediction for lipase 3 containing proteins in *A. limacinum* SR21

	Protein ID	SignalP	WolfPsort	DeepLOC	THMM
1.	Aurli_65985	OTHER	cyto	Cytoplasm	PredHel = 0
2.	Aurli_66074	OTHER	mito	Mitochondrion	PredHel = 0
3.	Aurli_70934	OTHER	extr	Cytoplasm|Lysosome/Vacuole	PredHel = 0
4.	Aurli_28098	SP(Sec/SPI)	extr	Extracellular	PredHel = 0
5.	Aurli_61176	OTHER	plas	Lysosome/Vacuole	PredHel = 4
6.	Aurli_62653	OTHER	extr	Plastid	PredHel = 0
7.	Aurli_757	OTHER	plas	Lysosome/Vacuole|Golgi apparatus	PredHel = 9
8.	Aurli_33542	SP(Sec/SPI)	extr	Extracellular|Lysosome/Vacuole	PredHel = 0
9.	Aurli_83242	OTHER	plas	Lysosome/Vacuole	PredHel = 7
10.	Aurli_34218	OTHER	mito	Cytoplasm|Lysosome/Vacuole	PredHel = 0
11.	Aurli_34357	OTHER	cyto_nucl	Cytoplasm|Lysosome/Vacuole	PredHel = 0
12.	Aurli_149169	OTHER	cyto_nucl	Cytoplasm	PredHel = 0
13.	Aurli_84442	OTHER	cyto_nucl	Cytoplasm|Mitochondrion|Lysosome/Vacuole	PredHel = 0
14.	Aurli_150216	SP(Sec/SPI)	mito	Extracellular	PredHel = 1
15.	Aurli_2999	OTHER	extr	Golgi apparatus	PredHel = 0
16.	Aurli_3000	OTHER	plas	Cell membrane	PredHel = 1
17.	Aurli_69819	OTHER	cyto	Lysosome/Vacuole	PredHel = 0
18.	Aurli_4322	OTHER	mito	Mitochondrion	PredHel = 3
19.	Aurli_136640	OTHER	extr	Lysosome/Vacuole	PredHel = 0
20.	Aurli_5590	SP(Sec/SPI)	extr	Extracellular|Lysosome/Vacuole	PredHel = 0
21.	Aurli_39720	OTHER	plas	Lysosome/Vacuole|Golgi apparatus	PredHel = 3
22.	Aurli_40553	OTHER	plas	Lysosome/Vacuole	PredHel = 9
23.	Aurli_8331	OTHER	mito	Mitochondrion	PredHel = 0
24.	Aurli_142142	OTHER	cyto	Cytoplasm	PredHel = 0
25.	Aurli_62170	OTHER	cyto	Lysosome/Vacuole	PredHel = 0
26.	Aurli_46041	OTHER	mito	Mitochondrion	PredHel = 0
27.	Aurli_13293	OTHER	plas	Lysosome/Vacuole|Golgi apparatus	PredHel = 4
28.	Aurli_145138	OTHER	plas	Extracellular	PredHel = 1
29.	Aurli_31293	SP(Sec/SPI)	extr	Extracellular|Lysosome/Vacuole	PredHel = 0
30.	Aurli_31294	OTHER	mito	Extracellular	PredHel = 0

**Fig. 1:**
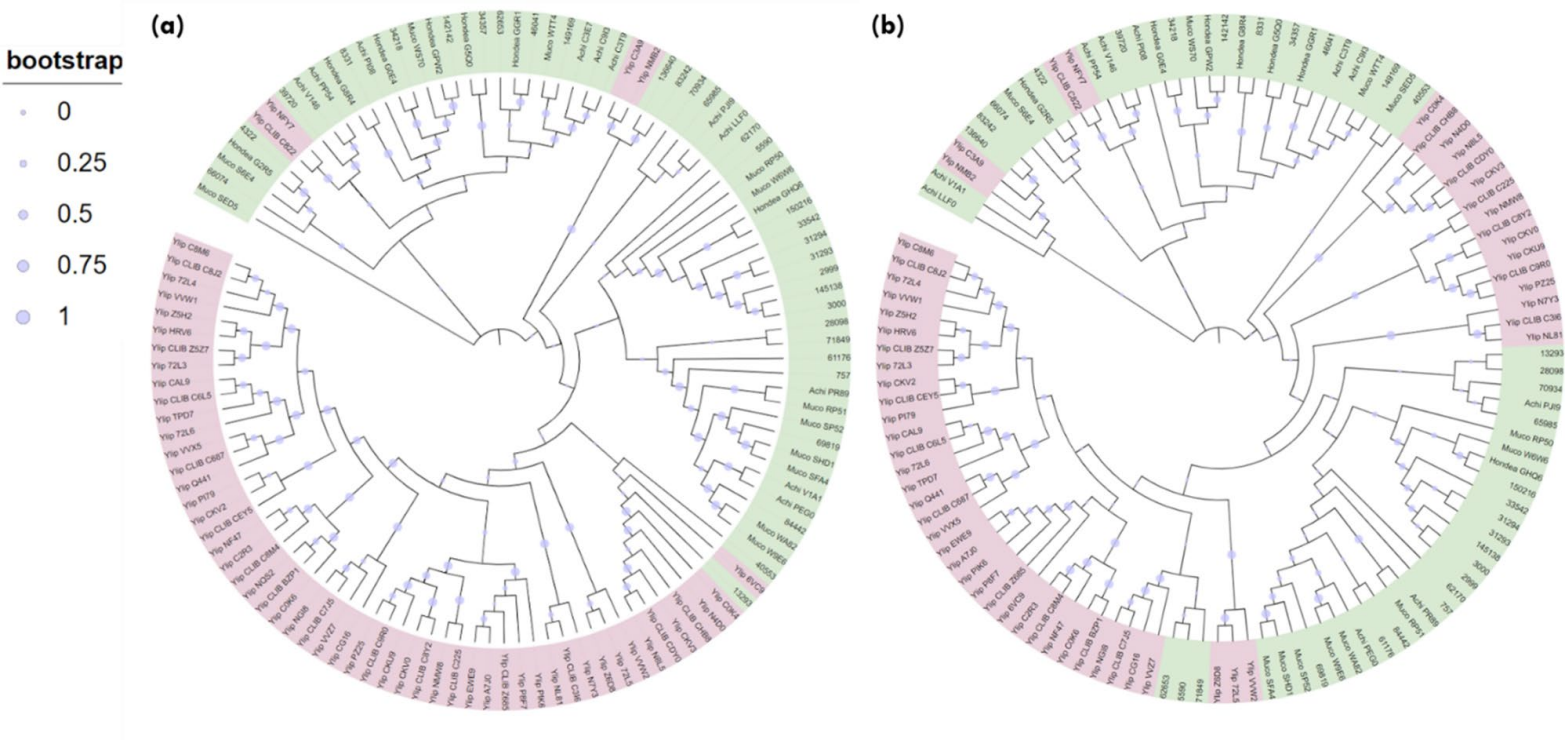
Phylogenetic tree constructed using (**a**) Maximum likelihood and (**b**) Neighbour joining method with 1000 bootstrapping. The bootstrapping values are depicted in the range of 0–1. Proteins corresponding to *A. limacinum* SR21 lipases are denoted with their IDs from JGI database. Green highlight represent thraustochytrid specific lipases whereas pink represents proteins from *Y. lipolytica*

**Fig. 2:**
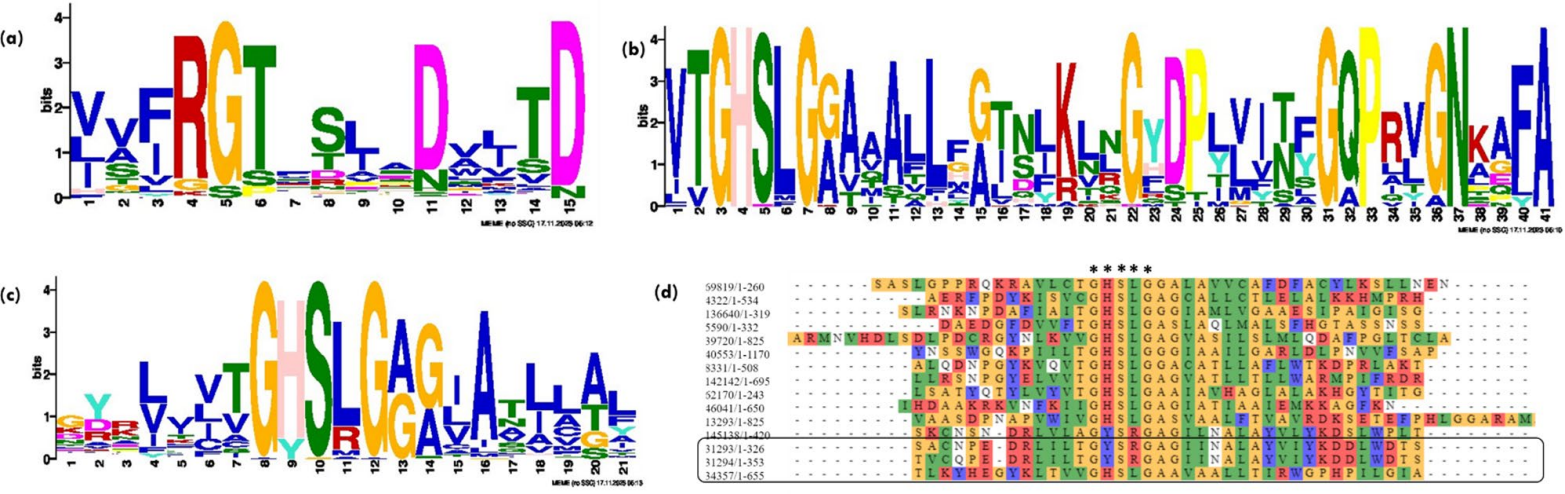
Motif enrichment analysis using MEME suite for Lipases from (**a**) thraustochytrid and *Y. lipolytica* (**b**) *Y. lipolytica* only (**c**) thraustochytrid only; (**d**) multiple sequence alignment for putative lipases from *A. limacinum* SR21 (GXSXG motifs are represented with ‘*’ and two selected lipases 31293 and 34357 with different motifs are highlighted)

Phylogenetic tree using the NJ- & ML- method was constructed (Fig. 1a & b) to determine the evolutionary relationship between these putative proteins. Both Maximum Likelihood (ML) and Neighbor-Joining (NJ) techniques were used to make sure the phylogenetic analysis was robust. NJ offers a computationally effective substitute for initial tree estimation and comparison, even if ML is typically more accurate. Cross-validation of tree topologies is made possible by using NJ results, which guarantees consistency and dependability in the results and makes it easier to compare them with earlier research that might have depended on NJ-based methodologies. Lipases for marine yeast *Y. lipolytica* are present in a distinct clade with high bootstrap support, while thraustochytrid-specific lipases are closely clustered together in separate clades of both NJ & ML-tree. This highlights that lipases from thraustochytrids are highly distinct in their sequence architecture from model yeast *Y. lipolytica.* In contrast to ML-tree, in the NJ method, a clade comprising a few lipases of *Y. lipolytica* were found to be clustered with thraustochytrid specific lipases, highlighting their origin from the common ancestor lineages. However, the maximum likelihood tree is usually preferred and more accurate over the NJ tree for computing evolutionary distances [40]. In Fig. 1b, few of the lipases from *A. limacinum* SR21 were found to be clustered with *Yarrowia* specific lipases, however the bootstrapping confidence was insignificant. Thraustochytrids are marine protists which are formerly considered as fungus as they share habitat and mode of nutrition [41]. However, they are more related to the marine diatoms and other stramenopiles of the Chromista kingdom [42]. To further gain insights into conserved sequence patterns in lipases, the MEME algorithm is used. It is widely used for the discovery of protein sequence motifs and the result is depicted in a form of logo plot. In a sequence logo plot, the height of each stack indicates the relative occurrence of the corresponding amino acid, while the color indicates the nature of the amino acid. Lipases have a characteristic GXSXG motif in which serine is the catalytic residue, and that makes them similar to serine proteases [43]. Figure 2a represents the sequence logo plot obtained for input lipases sequences, which shows conservation of RGT and D residues. The aspartic acid (D) residue was found to be part of the catalytic triad (Ser-His-Asp) of TAG lipases that forms hydrogen bonds with histidine and eventually allows deprotonation of serine residue [44]. Among the lipases identified in *Y. lipolytica*, the predicted motif comprises ‘GHSLG’ (Fig. 2b) with absolute conservation of His residue at the 2nd position of the pentapeptide. While the sequence logo plot depicted for *A. limacinum* SR21 lipases denote motif ‘GXSXG’ where histidine or tyrosine was found at the 2nd position (Fig. 2c), which is also evident from the sequence alignment displayed in Fig. 2d. The role of serine in this conserved GXSXG motif is well established, however if histidine has any significant role in lipase catalytic activity is not studied till date. The strict conservation of histidine in *Y. lipolytica* suggests its essential catalytic role, while the histidine/tyrosine variability in *A. limacinum* SR21 indicates possible functional or structural differences. Studying this substitution can provide insights into enzyme evolution, activity, and potential biotechnological applications. In this context, we selected two lipases 31293 & 34357 from *A. limacinum* SR21, where the former contain Tyr residue whereas the latter had His in the pentapeptide and predicted their structure using AlphaFold and performed docking analysis. 

### Structure analysis of thraustochytrid-specific lipases

**Fig. 3:**
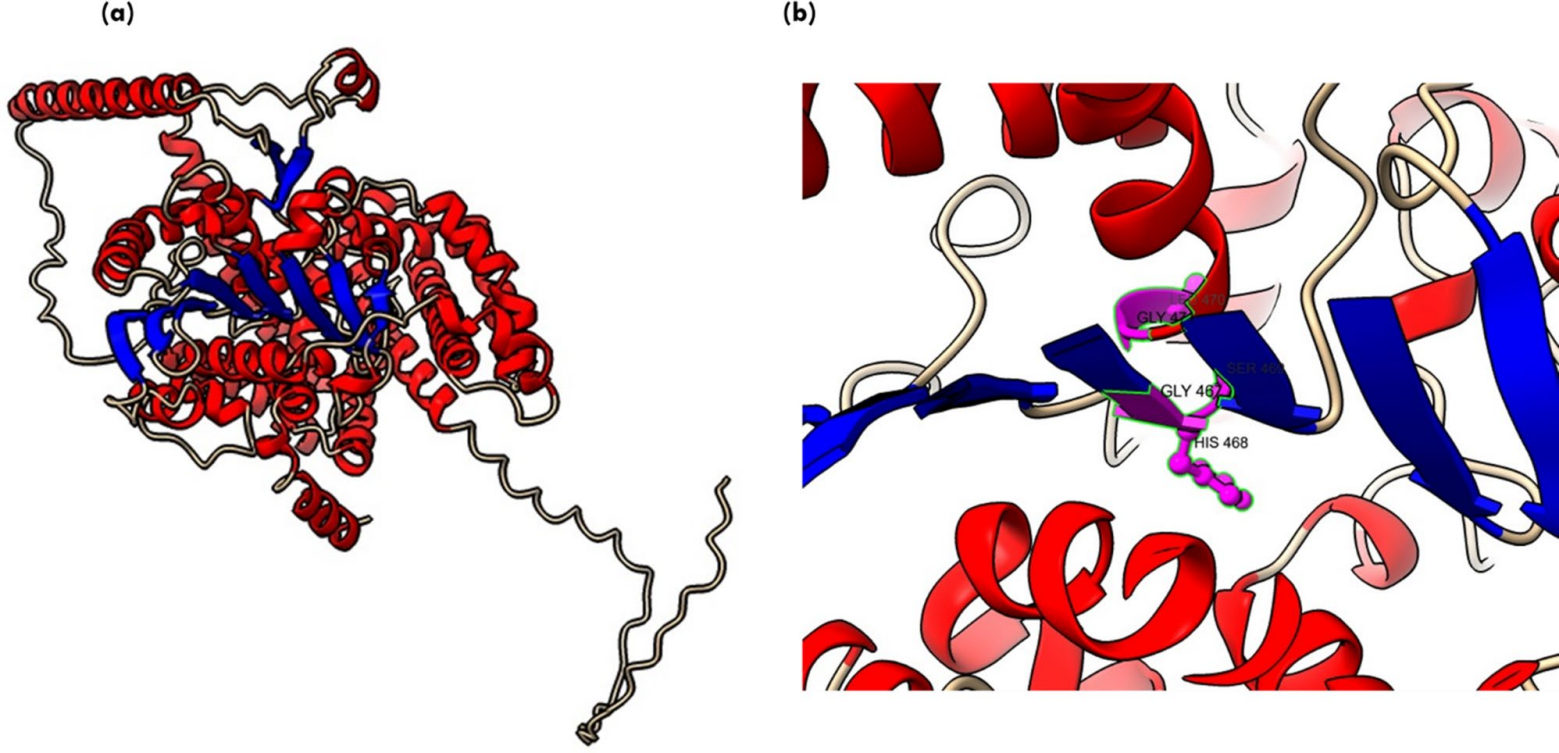
(**a**) Protein structure for cytoplasmic 34357 protein predicted using AlphaFold (helix is represented in red and β-sheets are represented in blue) (**b**) consensus motif residues GHSLG is represented in the form of red atoms

**Fig. 4:**
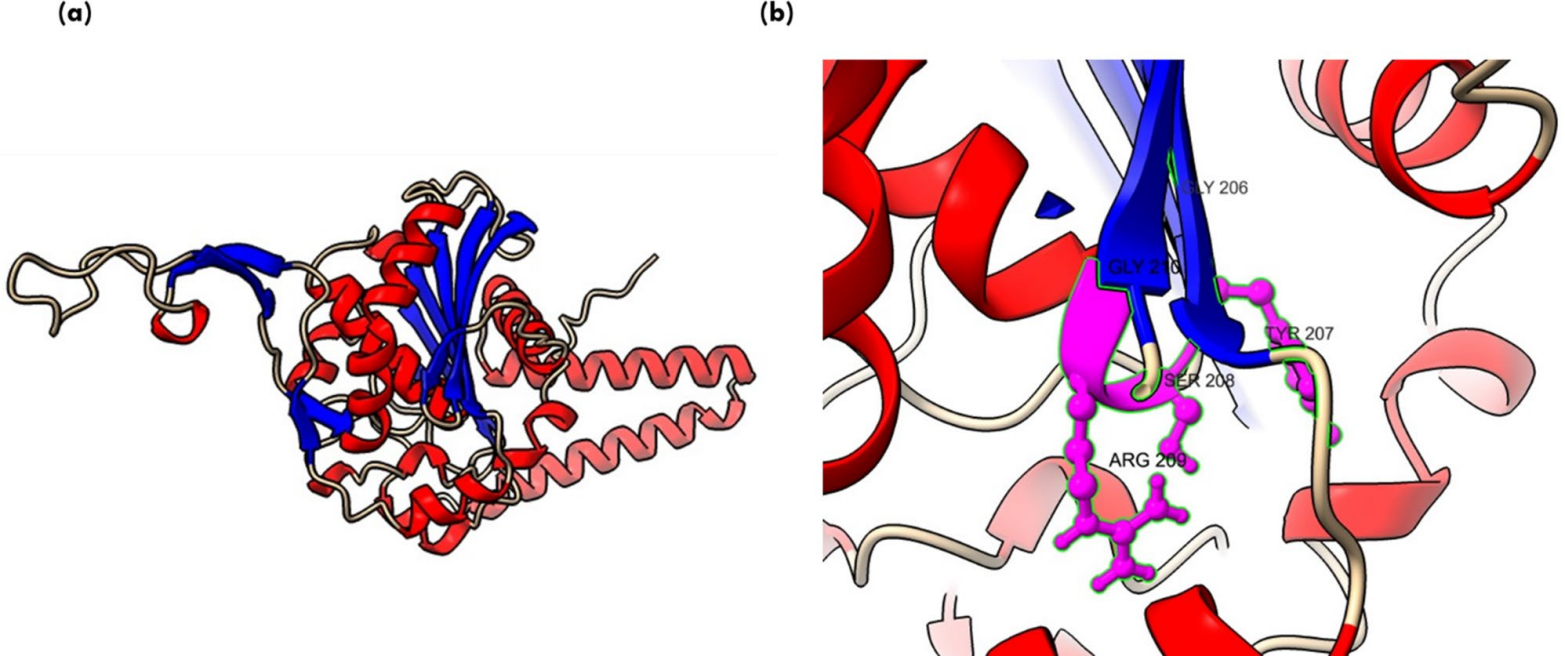
(**a**) Protein structure for secretory 31293 protein predicted using AlphaFold (helix is represented in red and β-sheets are represented in blue) (**b**) consensus motif residues GYSRG is represented in the form of red atoms

**Fig. 5:**
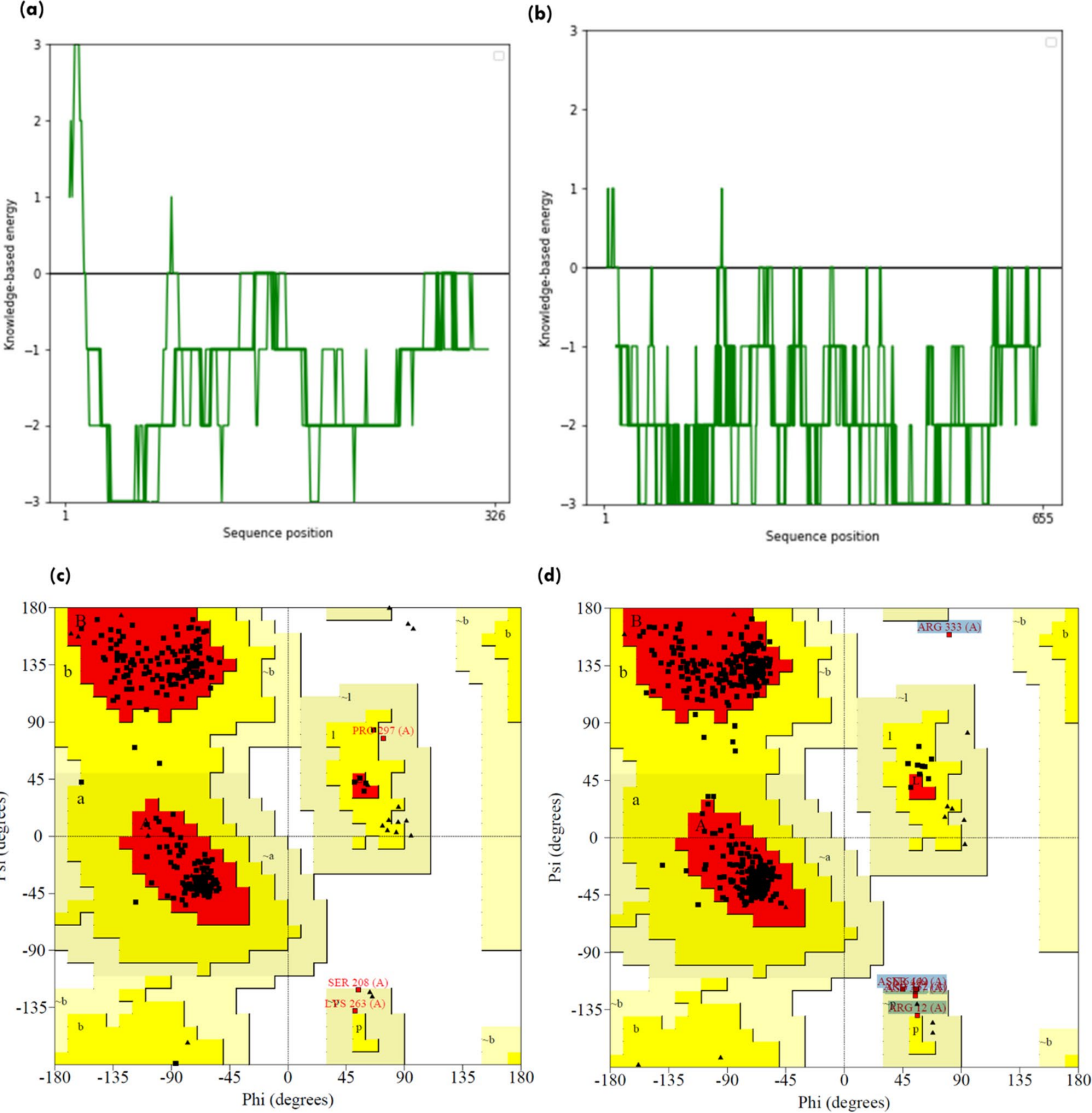
Structure quality assessment using (i) ProSA-web of residue scores for modelled protein (**a**) 31293 & (**b**) 34357; & (ii) Ramachandran plots depicting favored ϕ & ψ angles for 3D-structure of proteins (**c**) 31293 & (**d**) 34357

Lipases are a heterogeneous family of proteins with esterase activity and a characteristic α/β hydrolase fold. Their catalytic domain consists of parallel β-sheet structure (eight) connected by helices. The residues serine, aspartic acid and histidine form the catalytic triad, in which serine is part of conserved GXSXG motifs [45]. Protein 34357 was predicted to be cytosolic whereas protein 31293 was found to possess secretory signal and predicted to be extracellular. Previously, 145138 was reported to be the only extracellular lipase by Ishibashi et al., which lacked a signal peptide; however, a transmembrane domain was also predicted in the protein, further contrasting with the secretory nature [11]. Thus, protein structure was predicted for the extracellular lipase (31293) that possesses G**Y**SXG motif and cytosolic lipase (34357) that contains G**H**SXG residues using AlphaFold. AlphaFold combines evolutionary information from multiple sequence alignments, deep neural networks and advanced protein modeling techniques to predict 3D protein structures with high accuracy [22]. The two selected lipases are present in different clades in the phylogenetic tree (Fig. 1), which highlights their distinct sequence characteristics. For identification of the appropriate template required for modelling of these protein, sequence similarity search was done in the PDB database. Maximum similarity was obtained with the 1.7 Å resolution crystal structure of the Lip2 lipase (3O0D) from *Y. lipolytica* in its closed conformation, which was used as template for structure prediction [23]. For both proteins, five models were predicted and the model with higher pLDDT score was further selected for analysis. The pLDDT represents the model’s per-residue confidence on the scale of 0-100 and differs within regions for a single polypeptide. Thus, a region with high pLDDT represents well-predicted structure within a multi-domain enzyme [46]. The *A. limacnum* SR21 cytoplasmic lipase 31293 was 665 amino acids containing protein that was predicted to have a model pLDDT score of 63.3 for model = 5. The region with lipase 3 domain i.e., 364–476 was predicted with a pLDDT score greater than 70, thereby reflecting the high accuracy of the predicted structure (Supplementary Fig: 1). The protein structure of cytoplasmic lipase is represented in Fig. 3a, which contains 10 β-strands and multiple helices. Similar to other lipases, the β-strands are stacked together as parallel sheets and the serine containing motif GHSXG is present in sharp γ-turn between 5th β-strand and subsequent α-helix (Fig. 3b), thereby forming a ‘nucleophile elbow’ characteristic of α/β hydrolases [47]. Lipases are activated when exposed to oil-water interface, which is mediated by displacement of a lid domain hiding this nucleophilic elbow. These lid domains vary among species and comprise of either one or multiple helices or loops, which overlays the catalytic serine present in γ-turn. For 34357 the lid domain is composed of a single α-helix as depicted in Fig. 3b, whereas in Lip2 from *Y. lipolytica* loop is present in the lid domain [48]. In contrast to cytoplasmic lipases, the secretory 31293 is predicted with relatively higher pLDDT score (84.5) and comprises 11 β-strands (Fig. 4a). The structure of secretory lipase is highly distinct from 34357, where seven β-strands are stacked together whereas two strands are present looping out away from the main structure. The catalytic serine is present in the γ-turn between the 6th β-strand and subsequent α-helix which is covered by a helix lid domain (Fig. 4b). The modeled protein structures were further assessed using ProSa-web server that estimates the protein folding energy. The Z-score obtained for 31293 and 34357 were computed as -7.04 and − 10.26 respectively which were in the acceptable range of + 10 to -10 (negative being preferred). Z-score is determined by calculating each amino acid’s energy and comparing it to a known template structure [49]. The plot depicted in Fig. 5 (a & b) shows local model quality by plotting energies as a function of amino acid sequence position where positive values represent erroneous or problematic part of structure. The plot is smoothed by calculating the average energy over each 40-residue fragment. The statistical distribution of the possible combinations of the backbone dihedral angles ϕ and ψ is displayed in the Ramachandran plot (Fig. 5c & d). For 31293 protein, 96.9% residues were found in the most favored regions i.e., [A, B,L], while 2.4% residues were found in [a, b,l] region (additional allowed region). In contrast, 95.8% residues of 34357 protein were found in most favored region and 3.2% residues were allocated in additional allowed region. Additionally, three amino acid residues (Asn 6, Ala 11 & Gly 296) were predicted to be in unfavorable conformation (Supplementary Fig. 3). The Ramachandran plot’s permissible regions, in theory, indicate the potential values of the Phi/Psi angles for an amino acid (X) in an ala-X-ala tripeptide [50]. Structure validation can be done using the distribution of Phi/Psi values found in a protein structure [51]. For the dihedral angles, the Ramachandran plot shows the energetically permitted and prohibited regions. Dihedral angles are identified in the Ramachandran plot’s forbidden zones for low-quality homology models, which typically point to structural issues.

**Fig. 6:**
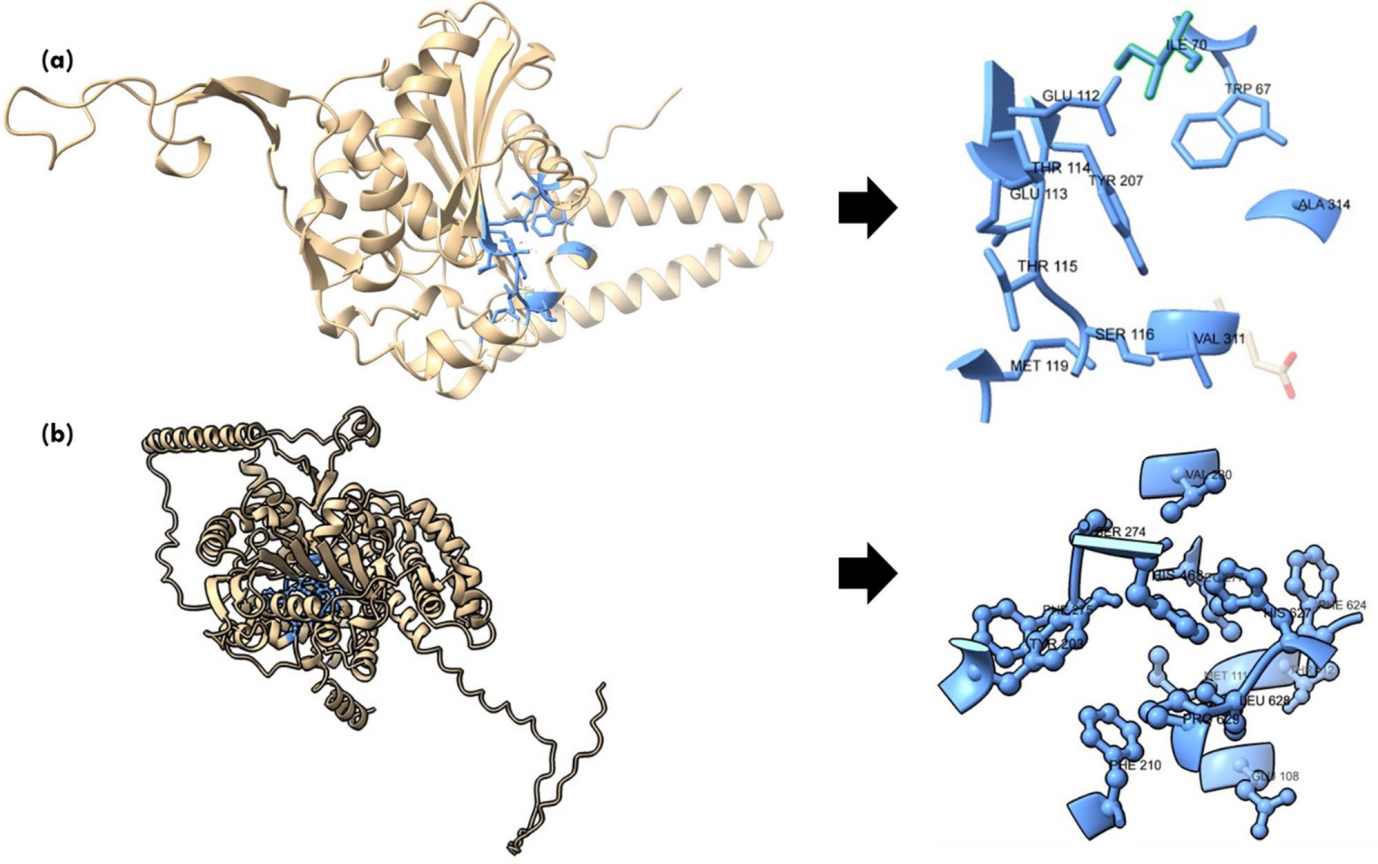
Ligand binding site prediction using PrankWeb for predicted protein structures of (**a**) 31293 & (**b**) 34357

The ligand binding site predicted for secretory lipase (31293) using PrankWeb is depicted in Fig. 6a. The pocket consists of 11 amino acid residues and has the highest score of 3.31. The amino acid residues that contribute to ligand binding sites in 31293 are W67, I70, E112, E113, T114, T115, S116, M119, **Y207**, V311 and A314. Ligand binding site for protein 34357 and the residues comprising the pocket are displayed in Fig. 6b. The ligand binding site with pocket score of 4.40 was further evaluated for its amino acid composition. The selected pocket in 34357 comprises of 14 amino acid residues namely E108, M111, T112, Y203, F210, S274, F275, L277, V280, **H468**, F624, H627, L628 & P629. As evident from the figure, that His residue which is part of the GHSXG motif and Tyr residue of GYSXG motifs of 34357 & 31293 are involved in the ligand binding pocket, thereby suggesting their significant role in lipase activity. Thus, the structural difference and variation among the ligand binding sites in lipases further reflect their differential catalytic efficiency.

## Docking and ligand binding analysis

Tripalmitoylglycerol (4RF) is a triacylglycerol moiety that is used as a ligand to perform docking analysis using AutoDock Vina. Ten distinct ligand poses were obtained from AutoDock docking simulations, with different binding energies. The ligand-protein interaction with the lowest Gibbs energy of binding (ΔG; kcal/mol) is listed in Table 2 and was further visualized using Discovery Studio for the identification of interacting partners. The cytoplasmic lipase with the His residue (34357) was found to have a lower ΔG i.e., -5.7 kcal/mol, while the secretory lipase had a relatively higher ΔG = -3.4 kcal/mol respectively. This suggests that the binding efficiency of protein with motif G**H**SXG is stronger than the lipase with G**Y**SXG. Docking parameters computed using DockThor resulted in a similar pattern (Supplementary_File2: Table 1). Further, to evaluate the effect of different protonation states of His468, DockThor was used and the ligand was docked to proteins with three His conformations (HisE, HisD, and HisP) (Table 3). HisE (Nε2 protonated) and HisD (Nδ1 protonated) show favorable binding, as evidenced by the negative binding affinity values and negative total energy values. These states appear to stabilize the ligand-protein complex. HisP (both Nδ1 and Nε2 protonated), however, results in a highly unfavorable interaction with a positive binding affinity and an extremely high total energy. The positively charged HisP may cause electrostatic repulsion with other residues or the ligand, destabilizing the binding interaction. This protonation state is likely not suitable for effective ligand binding.

**Table 2:** Docking parameters computed for selected lipases with tripalmitoylglycerol (4RF) as ligand using AutoDock Vina

Protein ID	Motif	Gibbs free energy (ΔG; kcal/mol)
34357	GHSLG	-5.7
31293	GYSRG	-3.4
31293(Y→H)	G**H**SRG	-3.4

**Table 3:** Docking parameters computed for 34357 with varying protonation States of His468 in GHSLG motif using DockThor

Protonation state of His468	Affinity (kcal/mol)	Total energy (kcal/mol)
HisE (Nε2 nitrogen, neutral)	-9.313	-20.192
HisD (Nδ1 nitrogen, neutral)	-8.282	-25.863
HisP (Nδ1 and Nε2, positive)	12.448	2888.786

**Fig. 7:**
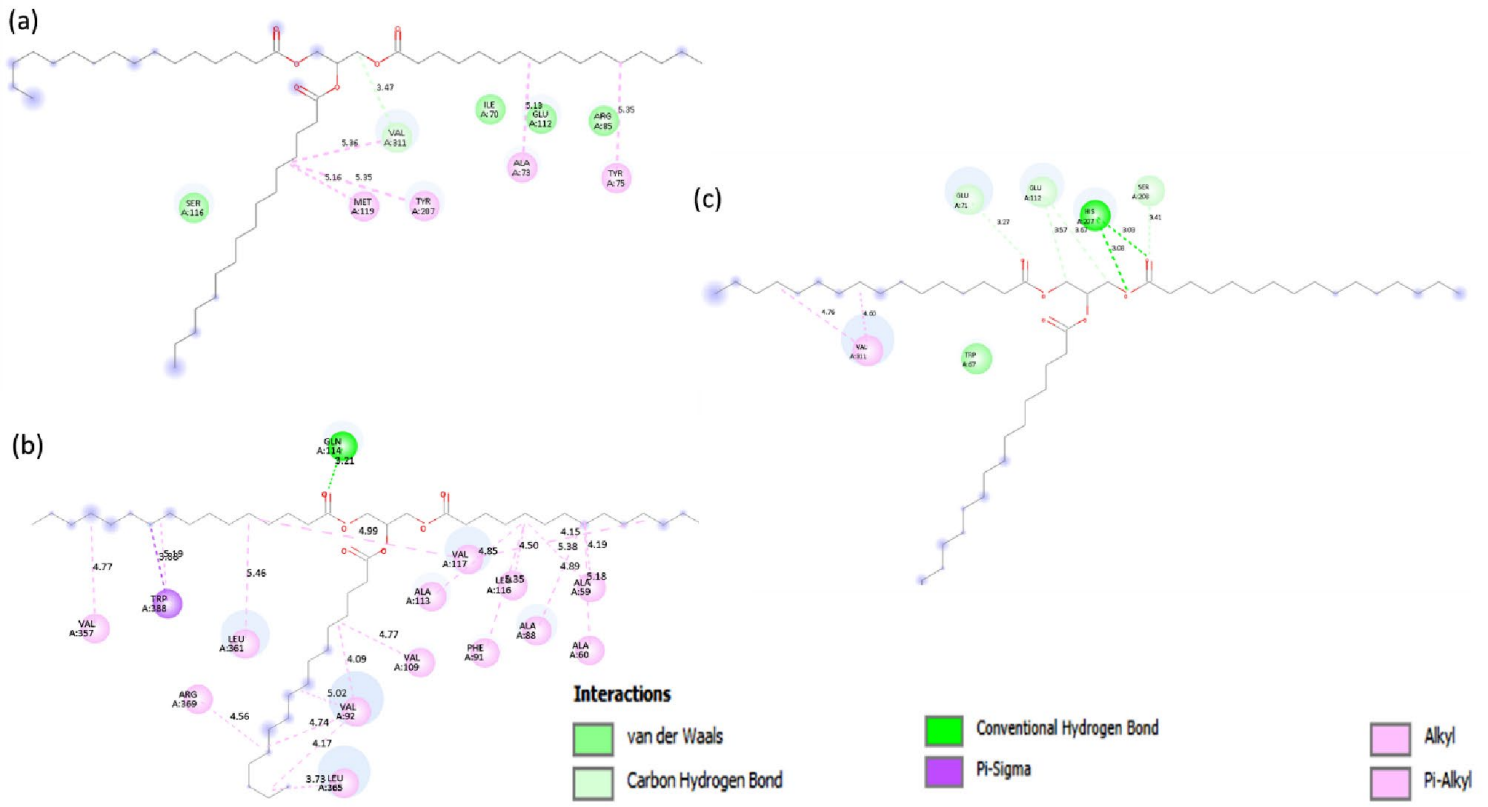
Ligand interaction diagram showing the molecular interactions between 4RF (tripalmitoylglycerol) and (**a**) 31293 (**b**) 34357 (**c**) 31293 (Y→ H)

In contrast to cytoplasmic lipase, the secretory protein 31293 has weak binding with the ligand i.e., ΔG = -3.4 kcal/mol. The interaction map for lipase 31293 (Fig. 7a) further highlights the difference in the interacting amino acid residues of the two proteins. Contrary to cytoplasmic lipase, the secretory lipase forms a weak C-H bond (3.47 Å) with Val311, which can stabilize hydrophobic ligands, like fatty acids or triglyceride substrates. The Tyr207 residue; within the G**Y**SXG motif of secretory lipase was found to interact with the hydrophobic fatty acid chain of the ligand using pi-alkyl bonds. This predicted interaction was similar to that observed for lipase in *Pseudomonas aeruginosa*, where residues like Ala and Met form the hydrophobic pocket and aromatic amino acids like Tyr participate in substrate binding and structural stability. The interaction diagram in Fig. 7b highlights the different kinds of bonds and bond lengths of ligand-amino acid residues of 34357 protein. A strong hydrogen bond (3.21 Å) was formed between the carbonyl (C = O) of the tripalmitoylglycerol molecule and amide group of Gln (Q114). In *Thermomyces lanuginosus* lipase (PDB: 1DT3), the catalytic triad (Gln, Ser, and His residues) form hydrogen bonds with the substrate, ensuring proper positioning for catalysis and strong binding with the ligand [52]. The amino acids Val357 (4.77 Å), Arg369 (4.56 Å), Leu365 (3.73 Å) form alkyl and pi-alkyl interactions and form a hydrophobic pocket that stabilizes the ligand molecules such as triglycerides. The amino acid composition of this hydrophobic pocket is similar to the one observed for pancreatic lipase (1LPB); which comprises Leu, Val, and Phe residues [53]. Aromatic residues like Trp and Phe stabilize binding through pi-pi and pi-sigma stacking interactions, similar to *Candida antarctica* lipase B (PDB: 1TCA) [54].

However, the interaction map demonstrated that the ligand-binding interactions were altered due to the Y207H substitution (Fig. 7c). His207 played a crucial role in substrate stabilization and activation by forming two hydrogen bonds (3.08 & 3.03 Å) with the ester bond of triacylglycerol (TAG). His207 also contributed to catalysis by facilitating the deprotonation of Ser208, thereby activating it for nucleophilic attack on the ester carbonyl of TAG. Additionally, Glu116 formed C-H bonds (3.57 & 3.67 Å) with the glycerol backbone, ensuring proper substrate positioning in the active site. These findings suggest that His207 enhances substrate orientation and activation in the lipase active site, reinforcing the importance of catalytic triad residues in lipase function. Further experimental validation could provide deeper insights into the functional significance of this substitution in lipase activity.

## Conclusions

In conclusion, this study identifies that marine heterotrophic thraustochytrids have multiple secretory, cell-bound and intracellular lipases that allow them to thrive on oil substrates. As observed from the phylogenomic analysis of these lipases, thraustochytrid-specific lipases form distinct clades from those of *Y. lipolytica*, suggesting evolutionary divergence and potential functional differences. Motif enrichment analysis identified variations in the GXSXG motif, where lipases in *Y. lipolytica* have strictly conserved His at the second position, whereas thraustochytrid lipases displayed variability between His and Tyr, highlighting possible differences in catalytic mechanisms. Structural modeling of two selected lipases (31293 & 34357) further emphasized these variations. The cytoplasmic His-containing lipase (34357) demonstrated a stronger binding affinity (ΔG = -5.7 kcal/mol) compared to the secretory Tyr-containing lipase (31293, ΔG = -3.4 kcal/mol), suggesting that His substitution could enhance ligand interaction and enzymatic efficiency. Docking analysis indicated that His207 forms critical hydrogen bonds (3.08 & 3.03 Å) to stabilize the substrate in the active site, facilitating Ser208 deprotonation and nucleophilic attack on TAG. Additionally, Glu116 stabilizes the glycerol backbone, ensuring optimal substrate positioning. These findings suggest that His207 plays a crucial role in lipase activity by enhancing substrate orientation and activation. The results of this study have important commercial implications for the industrial application of lipases. Lipases are widely used in biotechnology, pharmaceuticals, food processing, biofuel production, and detergent formulations. Enhancing enzyme efficiency by optimizing active-site residues, such as substituting Tyr with His, could lead to lipases with improved catalytic activity, higher substrate specificity, and better thermostability. Thraustochytrids can be an advantageous host for commercial production of these lipases as they are osmotolerant and leading producers of value-added product ω-3 fatty acids, thereby making the process more economically viable. Furthermore, the structural insights gained from this study can aid in enzyme engineering for tailored applications, allowing for rational design of highly efficient, stable, and commercially viable lipases. Additionally, such in silico studies could help us design non-native *sn-2* specific lipases, which are of utmost importance for the production of structured lipids but are rarely reported for microbes and none have been commercialized to date. By leveraging these insights, biotechnological applications of lipases can be optimized for higher efficiency, cost-effectiveness, and sustainability.

## Electronic supplementary material

Below is the link to the electronic supplementary material.

Supplementary Material 1

## Data Availability

The datasets used and/or analyzed during the current study such as predicted protein structures are available from the corresponding author on reasonable request.

## Abbreviations

E.C. number: Enzyme commission number
ΔG: Gibb’s free energy
TAG: Triacylglycerol
MEME: Multiple Em for motif elicitation
