# Supplementary material for: Structural insights into thraustochytrid-specific lipases using alphafold to identify the role of GXSXG motif
Source: BMC Biotechnol. 2025 May 5;25:32. doi: 10.1186/s12896-025-00972-8 (PMC12054267; doi:10.1186/s12896-025-00972-8)
Supplement: Supplementary file 1 — Supplementary Material 1 [file 12896_2025_972_MOESM1_ESM.docx]

**Supplementary Fig. 1: pLDDT score plot for cytoplasmic TAG lipase (34357) predicted using AlphaFold.**


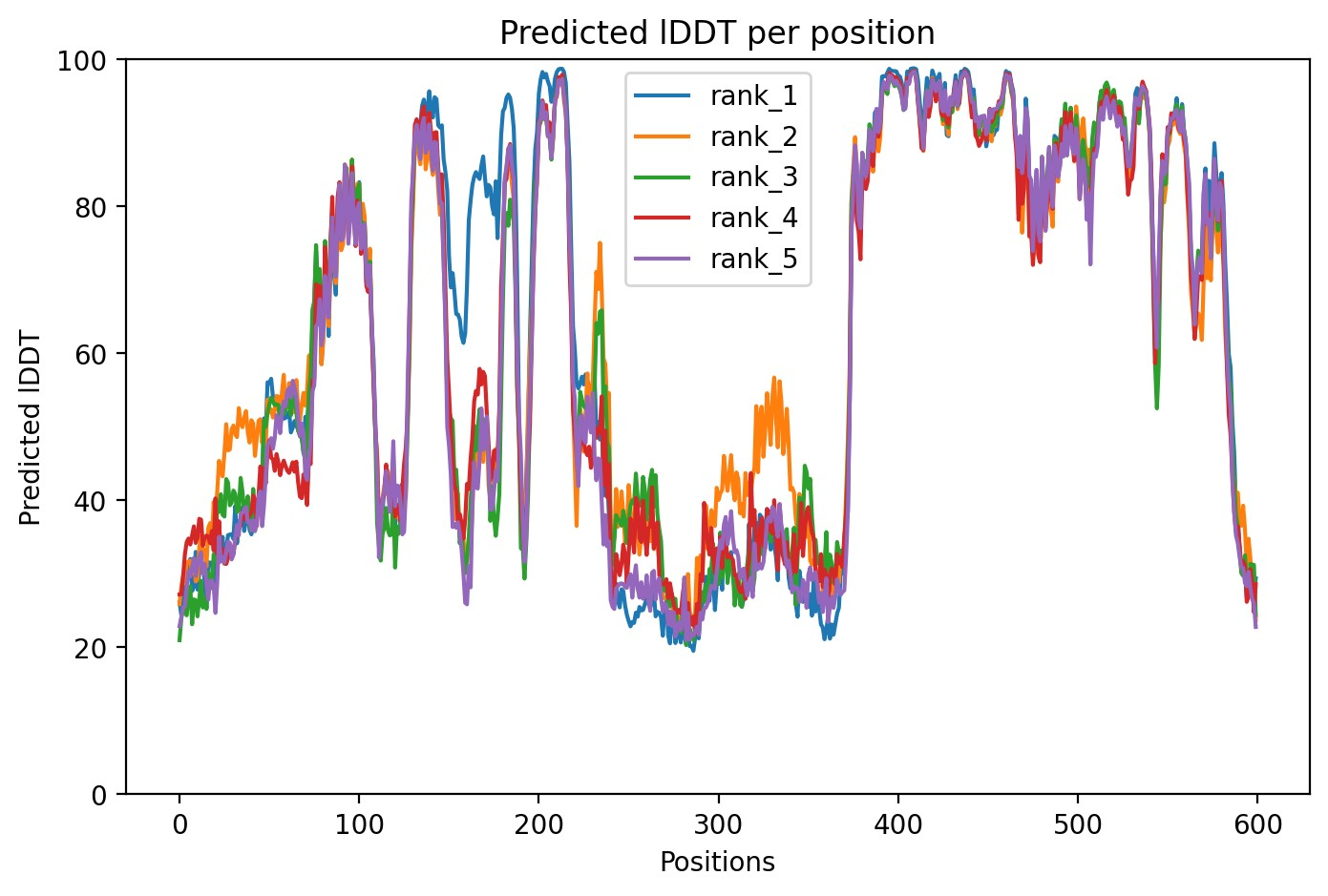


**Supplementary Fig. 2: pLDDT score plot for extracellular TAG lipase (31293) predicted using AlphaFold.**


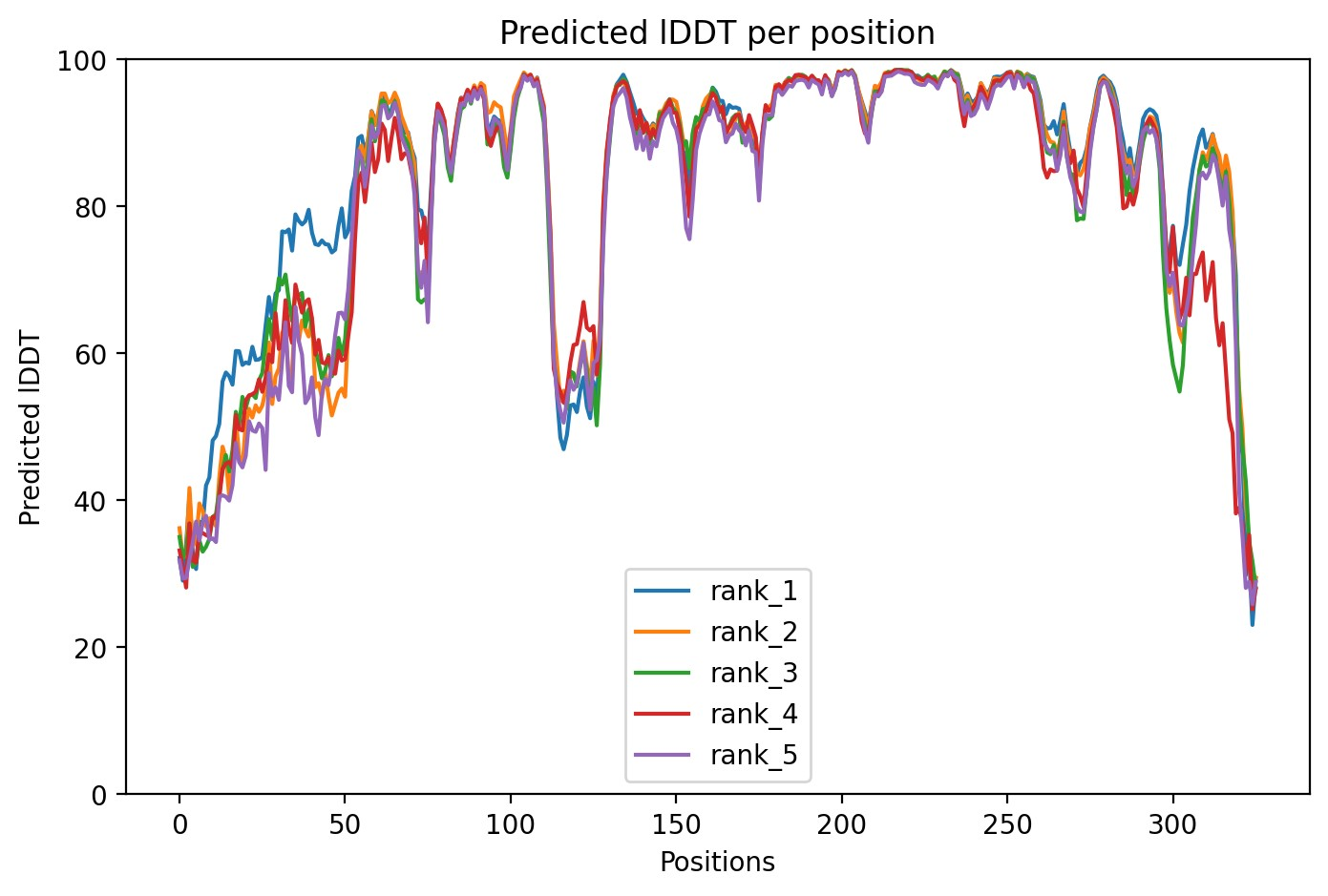


**Supplementary Fig. 3: Ramachandran plot for amino acids of protein 34357 depicted amino acids with unfavorable conformations.**


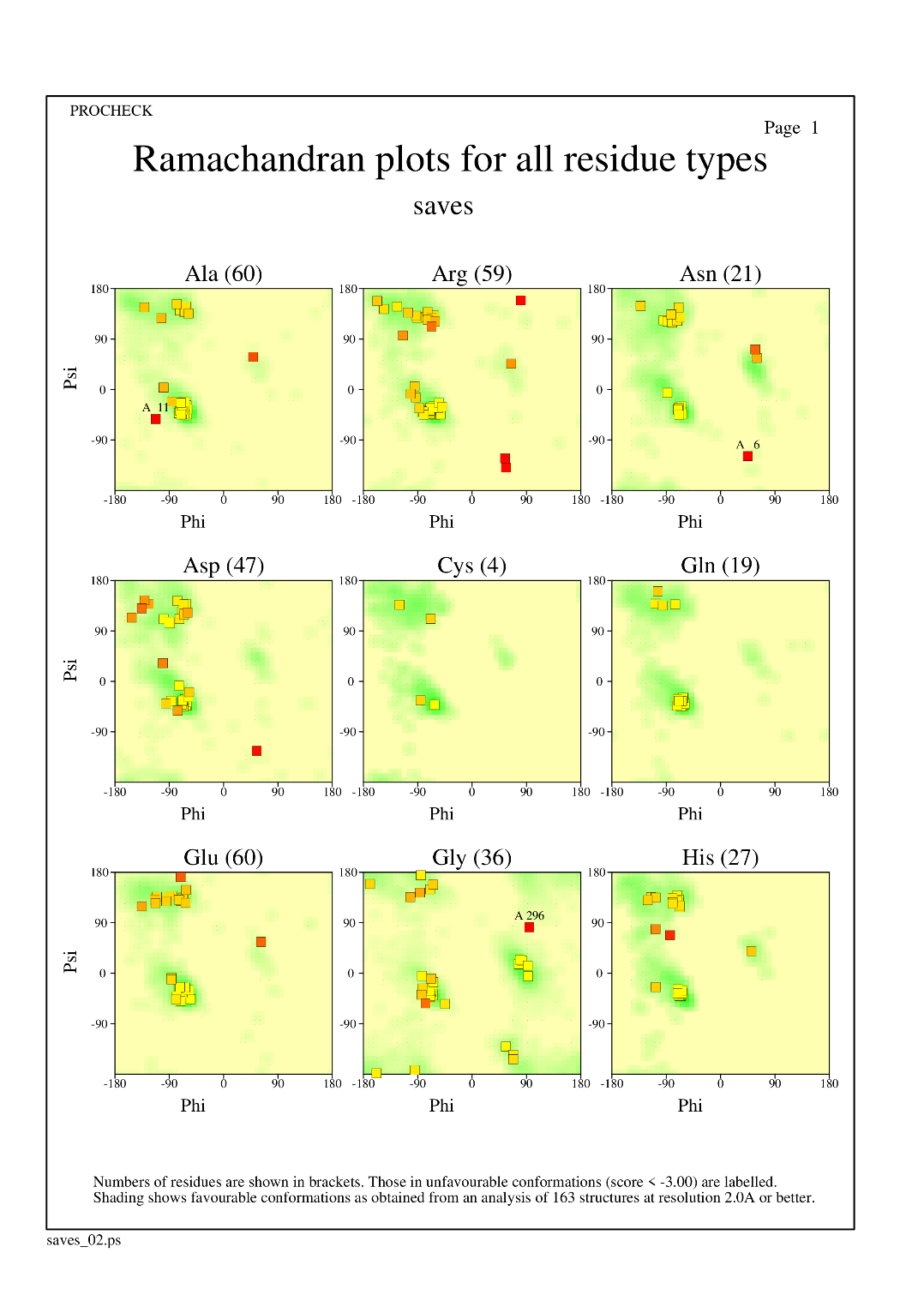


**Supplementary Table 1: Docking parameters computed for selected lipases with tripalmitoylglycerol (4RF) as ligand using DockThor.**

| Protein_ID | Motif | Affinity (kcal/mol) | Total Energy (kcal/mol) |
| --- | --- | --- | --- |
| 34357 | GHSLG | -9.313 | -20.192 |
| 31293 | GYSRG | -8.282 | 2.894 |
